# Effect of Diabetes Mellitus on Acute Kidney Injury after Minimally Invasive Partial Nephrectomy: A Case-Matched Retrospective Analysis

**DOI:** 10.3390/jcm8040468

**Published:** 2019-04-05

**Authors:** Na Young Kim, Jung Hwa Hong, Dong Hoon Koh, Jongsoo Lee, Hoon Jae Nam, So Yeon Kim

**Affiliations:** 1 Department of Anesthesiology and Pain Medicine, Anesthesia and Pain Research Institute, Yonsei University College of Medicine, 50-1 Yonsei-ro, Seodaemun-gu, Seoul 03722, Korea; knnyyy@yuhs.ac (N.Y.K.); HJNAM90@yuhs.ac (H.J.N.); 2 Department of Policy Research Affairs National Health Insurance Service Ilsan Hospital, 100 Ilsan-ro, Ilsandong-gu, Goyang, Gyeonggi-do 10444, Korea; jh_hong@nhimc.co.kr; 3 Department of Urology, Konyang University College of Medicine, 158 Gwanjeodong-ro, Daejeon 35365, Korea; urodhkoh@kyuh.ac.kr; 4 Department of Urology and Urological Science Institute, Yonsei University College of Medicine, 50-1 Yonsei-ro, Seodaemun-gu, Seoul 03722, Korea; JS1129@yuhs.ac

**Keywords:** diabetes mellitus, acute kidney injury, nephrectomy, minimally invasive surgical procedures, risk factors

## Abstract

Postoperative acute kidney injury (AKI) is still a concern in partial nephrectomy (PN), even with the development of minimally invasive technique. We aimed to compare AKI incidence between patients with and without diabetes mellitus (DM) and to determine the predictive factors for postoperative AKI. This case-matched retrospective study included 884 patients with preoperative creatinine levels ≤1.4 mg/dL who underwent laparoscopic or robot-assisted laparoscopic PN between December 2005 and May 2018. Propensity score matching was employed to match patients with and without DM in a 1:3 ratio (101 and 303 patients, respectively). Of 884 patients, 20.4% had postoperative AKI. After propensity score matching, the incidence of postoperative AKI in DM and non-DM patients was 30.7% and 14.9%, respectively (*P* < 0.001). In multivariate analysis, male sex and warm ischemia time (WIT) >25 min were significantly associated with postoperative AKI in patients with and without DM. In patients with DM, hemoglobin A1c (HbA1c) >7% was a predictive factor for AKI, odds ratio (OR) = 4.59 (95% CI, 1.47–14.36). In conclusion, DM increased the risk of AKI after minimally invasive PN; male sex, longer WIT, and elevated HbA1c were independent risk factors for AKI in patients with DM.

## 1. Introduction

Partial nephrectomy (PN) is the current gold standard treatment for small, localized renal tumors owing to reduced risk of acute and chronic kidney dysfunction compared with radical nephrectomy [1,2]. Nevertheless, the incidence rate of acute kidney injury (AKI) after PN is 12%–54% depending on the definition of AKI [2,3,4]. In case of robot assisted PN, the incidence of postoperative AKI was reported as 24%–27% [5,6]. Therefore, postoperative AKI remains a concern in minimally invasive PN, as parenchymal mass reduction and/or ischemic injury due to vascular clamping cannot be avoided [4].

Diabetes mellitus (DM) has an increasing global prevalence and is the leading cause of chronic kidney disease [7]. Moreover, patients with DM are at an increased risk of acute kidney dysfunction throughout their lifetime [8]. Furthermore, DM is a recognized risk factor for AKI in the postoperative setting, such as in cardiac [9] and non-cardiac surgeries [10,11]. Previous studies investigating risk factors for AKI in all patients who underwent PN did not distinguish the open technique from the minimally invasive approach [3,4,12,13,14,15]. Only one study evaluated the effect of warm ischemia time (WIT) on AKI in robot-assisted laparoscopic PN [5]. Therefore, data on risk factors for AKI after minimally invasive PN are limited. Furthermore, no study has determined whether patients with DM have an increased risk of AKI after minimally invasive PN. The present study aimed to compare the AKI incidence between patients with and without DM and to investigate the predictive factors for AKI in these patients after laparoscopic and robot-assisted laparoscopic PN. 

## 2. Material and Methods

### 2.1. Patients

**Figure 1:**
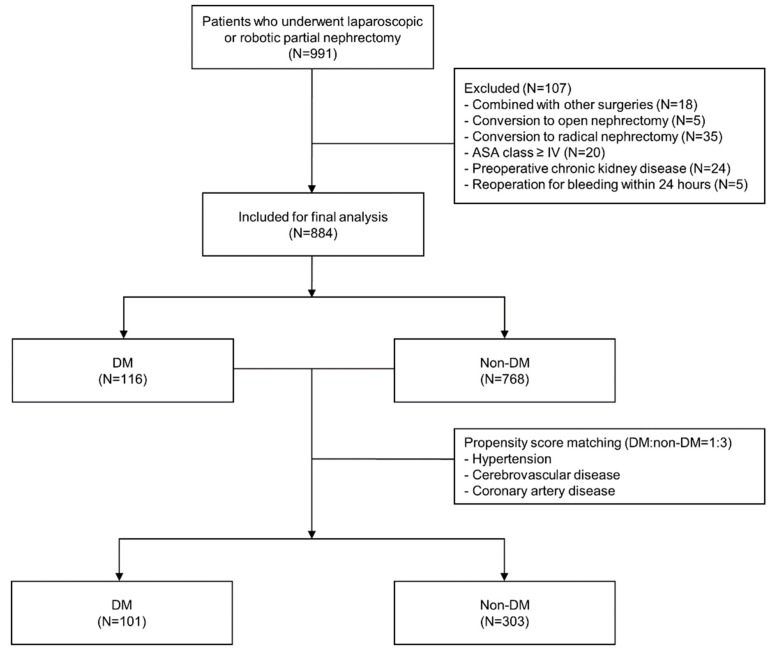
Flow diagram of patient selection. ASA—American Society of Anesthesiologists; DM—diabetes mellitus.

This case-matched retrospective analysis was performed after obtaining approval from the institutional review board and hospital research ethics committee (Yonsei University Health System, Seoul, Korea; IRB protocol No. 4-2018-0678, approved at August 29, 2018) with informed consent form from the patients being waived off. A total of 991 patients who underwent laparoscopic or robot-assisted laparoscopic PN between December 2005 and May 2018 were identified from the electronic medical records of a single institution, of which 58 were excluded owing to the type of operation; specifically, 18 patients underwent other combined procedures, whereas 5 and 35 patients were converted to open and radical nephrectomy, respectively. Furthermore, 20 patients with an American Society of Anesthesiologists (ASA) physical status ≥IV, 24 patients with an underlying chronic kidney disease or preoperative creatinine level >1.4 mg/dL, and 5 patients who underwent reoperation because of bleeding within 24 hours postoperatively were excluded from the analysis. Finally, 884 patients were identified, and propensity score matching was performed to match patients with and without DM in a 1:3 ratio (101 and 303 patients, respectively). In propensity score matching, hypertension, cerebrovascular disease, and coronary artery disease were used as covariates (Figure 1).

Standard general anesthesia was provided to all patients. Propofol, remifentanil, and rocuronium were used for anesthesia induction, whereas sevoflurane or desflurane and remifentanil were used for anesthesia maintenance. Administered colloid solution was 6% hydroxyethyl starch 130/0.4 (Volulyte^®^ or Voluven^®^, Fresenius-Kabi, Seoul, Korea). Laparoscopic or robot-assisted laparoscopic PN was performed in accordance with our institution’s protocol [16]. Tumor bed closure was performed using renorrhaphy with absorbable synthetic braided sutures or absorbable barbed sutures according to the surgeon’s preference. In cases with calyceal opening, additional suturing was also performed to maintain watertightness.

### 2.2. Data Collection

All data were collected from electronic medical records. Demographic data included age, sex, body mass index, ASA physical status, and underlying diseases, such as diabetes controlled with oral medication or insulin, hypertension, cerebrovascular disease, and coronary artery disease. Cerebrovascular disease was defined as transient ischemic attack, stroke, history of carotid artery stent, and cerebral hemorrhage. Preoperative laboratory data included creatinine, hematocrit, and hemoglobin (Hb) A1c levels. Operative data included the type of operation, operative time, WIT, volume of intraoperatively administered fluid, intraoperative use of a colloid solution or packed red blood cells, and intraoperative urine output. Data on serum creatinine level and estimated glomerular filtration rate (eGFR) were collected before surgery, immediately after surgery, and at 1 day, 2 days, 1 month, and 3 months after surgery. The eGFR value was calculated using the Chronic Kidney Disease Epidemiology Collaboration equation.

### 2.3. Primary and Secondary Outcomes

The primary outcome was comparison of AKI incidence between patients with and without DM after laparoscopic and robot-assisted laparoscopic PN. AKI was defined as an absolute increase in serum creatinine level by ≥0.3 mg/dL or ≥50% increase from the preoperative value within the first 48 h after surgery [17]. AKI was further categorized into three stages according to the acute kidney injury network classification: Stage 1, an increase in serum creatinine level ≥0.3 mg/dL or ≥150%–200% (1.5–2-fold) from the baseline value; stage 2, an increase in serum creatinine level >200%–300% (2–3-fold) from the baseline value; and stage 3, an increase in serum creatinine level >300% (3-fold) from the baseline value [17]. Additionally, we investigated predictive factors for AKI in patients with and without DM after laparoscopic and robot-assisted laparoscopic PN as the secondary outcome.

### 2.4. Statistical Analysis

Continuous variables are presented as mean (SD), whereas categorical variables are expressed as the number of patients in percentage. Continuous and categorical variables were evaluated using independent *t*-test and chi-squared test, respectively. Propensity score matching using a 1:3 ratio was performed to adjust the baseline characteristics of patients with and without DM. Multivariate logistic regression was employed to identify risk factors for AKI in patients with and without DM. A *P* value <0.05 was considered statistically significant. All statistical analyses were performed using SAS software version 9.4 (SAS Institute, Cary, NC, USA).

## 3. Results

In the analysis of all patients (*N* = 884) prior to matching, the incidence rate of AKI after minimally invasive PN was 20.4%, and the following variables were identified as independent risk factors for AKI (Table 1): Male sex, DM, longer operative duration, WIT >25 min, and higher intraoperative urine output. 

**Figure 2:**
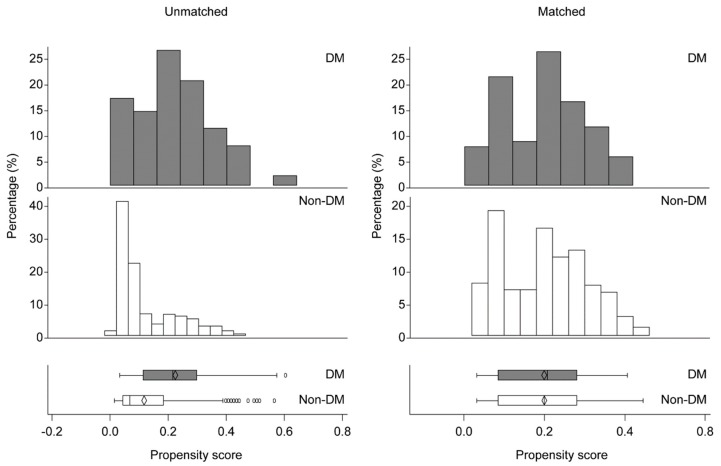
Distribution of propensity scores of patients with and without diabetes mellitus (DM) before and after matching. DM, Diabetes mellitus.

Among patients with a concomitant disease, only DM was shown to be a risk factor for AKI. Hence, we decided to investigate the effect of DM on AKI using propensity score matching. Prior to matching, the number of patients with DM who were older and had hypertension, cerebrovascular disease, and coronary artery disease was higher than that of patients without DM (Table 2). However, after matching, the demographic characteristics of patients with and without DM were similar except for the ASA physical status (Table 2) and the distribution of patients with and without DM was fairly uniform (Figure 2).

**Figure 3:**
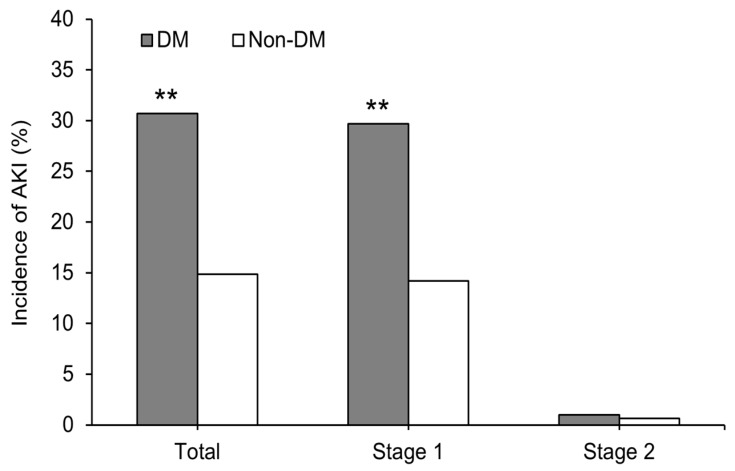
Incidence of acute kidney injury after minimally invasive partial nephrectomy according to the acute kidney injury network criteria. **P < 0.001 versus non-DM patients. DM, Diabetes mellitus; AKI, acute kidney injury; stage 1, increase in the serum creatinine level ≥0.3 mg/dL or ≥150%–200% (1.5–2-fold) from baseline; stage 2, increase in the serum creatinine level >200%–300% (2–3-fold) from baseline.

After matching, the incidence rate of postoperative AKI was significantly higher in patients with DM than in those without DM (total: 30.7% vs. 14.9%, *P* < 0.001; stage 1: 29.7% vs. 14.2%, *P* < 0.001; stage 2: 1.0% vs. 0.7%, *P* > 0.999) (Figure 3). No patient developed stage 3 of AKI. 

Results of the univariate and multivariate analyses of risk factors for AKI in patients with and without DM are summarized in Table 3. Male sex and WIT >25 min were determined to pose a significantly higher risk of postoperative AKI in patients with and without DM. In patients with DM, preoperative HbA1c >7% was a predictive factor for AKI: Odds ratio (OR) = 4.59 (95% confidence interval (CI), 1.47–14.36). When patients were classified according to DM and sex, the probabilities of AKI were as follows: Females without DM, OR = 1; females with DM, OR = 0.95 (95% CI: 0.16–5.59); males without DM, OR = 4.12 (95% CI: 1.32–12.86); and males with DM, OR = 14.46 (95% CI: 4.62–45.25) (Table 4).

**Figure 4:**
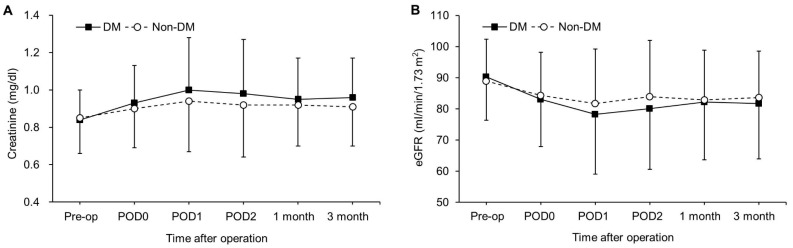
Changes in the serum creatinine level (**A**) and estimated glomerular filtration rate (**B**) until three months postoperatively. Values are presented as a mean (SD). No significant differences were observed between patients with and without DM at each time point. DM, Diabetes mellitus; eGFR, estimated glomerular filtration rate; Pre-op—preoperatively; POD0—postoperative day 0 (immediately after the operation); POD1—postoperative day 1—POD2, postoperative day 2.

The perioperative serum creatinine level and eGFR until three months after surgery are shown in Figure 4. Although no significant difference in serum creatinine level and eGFR was observed between patients with and without DM at each time point, there were significant intergroup differences over time (*P*_Group×Time_ = 0.016 and 0.026, respectively). 

## 4. Discussion

This is the first retrospective case-matched study to clarify whether patients with DM have an increased risk of AKI and evaluate risk factors for AKI in patients with and without DM after minimally invasive PN. The main findings were as follows: (1) DM was a strong predictive factor for AKI after minimally invasive PN. (2) The incidence rate of postoperative AKI was significantly higher in patients with DM than in those without DM, even after adjustment for other factors. (3) Male sex and WIT >25 min were predictive factors for AKI in both patients with and without DM. (4) Preoperative HbA1c >7% was a predictive factor for AKI in patients with DM

Although PN is associated with a reduced risk of postoperative AKI compared with radical nephrectomy owing to being a nephron-sparing surgery [15], the incidence rate of AKI after PN has been reported to be 12%–54% [2,3,4]. Although AKI incidence varies depending on the definition used, this incidence rate is definitely higher than that after non-cardiovascular surgery (1%–11%) [10,11]. In our study, the incidence rate of AKI in 884 patients who underwent laparoscopic or robot-assisted laparoscopic PN was 20.4%, which is comparable to the previously reported incidence rate of 24%–27% after robot-assisted laparoscopic PN [5,6]. Postoperative AKI is known as a high-risk factor for new-onset chronic kidney disease and is associated with increased morbidity and mortality after nephrectomy and non-cardiovascular surgery [10,11,18,19]. Therefore, attention should be paid to AKI after PN even with the development of the minimally invasive technique, and identification of patients at risk before surgery is important.

Several studies have investigated predictive factors for AKI after PN, which included open and minimally invasive techniques [3,4,12,13,14,15]. Longer WIT and operative duration as well as patient-related factors, such as old age, male sex, obesity, impaired preoperative kidney function, and history of hypertension were identified as risk factors for postoperative AKI [3,4,12,13,14,15,20,21]. In our study, which analyzed risk factors after minimally invasive PN only, WIT >25 min, longer operative duration, and patient-related factors, such as male sex, presence of DM, and high intraoperative urine output were revealed to be independent predictors of postoperative AKI. A previous study that included intraoperative urine output as one of the variables for analysis showed that a low urine output is a risk factor for AKI after PN [3]. The high urine output in the present study could be attributed to the use of diuretics typically prescribed by surgeons in difficult cases, which could be the reason for the different result in our study. As urine output in anesthetized patients has been proven to be a poor indicator of fluid balance and is not a predictive factor for AKI after general surgery [11], further studies on the relationship between intraoperative urine output and AKI after PN are required to establish a definite conclusion.

Although our study is the first to identify DM as a risk factor for AKI in PN, previous studies have already reported DM as a risk factor for AKI after general surgeries [10,11]. One study, which enrolled 7564 non-cardiovascular surgical patients and applied the same definition of AKI that we used, reported an OR of 1.51 (95% CI, 1.11–2.06) for AKI development in patients with DM [11]. In comparison, the OR for AKI development in patients with DM in our study was 2.85 (95% CI, 1.71–4.74). Moreover, even after adjustment for other factors by propensity score matching, the incidence rate of AKI was significantly higher in patients with DM (30.7%) than in those without DM (14.9%). Several experimental studies have indicated the susceptibility to renal ischemia/reperfusion (I/R) injury in DM [22,23,24]. Compared with the nondiabetic kidney, the diabetic kidney exhibited severe damage, including increased tubular cell apoptosis, tubulointerstitial fibrosis, and decreased tubular proliferation after renal I/R [22,23]. Moreover, reperfusion after renal ischemia was markedly delayed in diabetic mice than that in nondiabetic mice [24]. Therefore, the combination of these factors might have made patients with DM more susceptible to AKI in our study.

WIT is recognized as a strong contributing factor for AKI after PN [3,4,5,12,13,20,21]. WIT >25 min results in irreversible damage that is diffusely distributed throughout the operated kidney, even at six months after PN [25]. Porpiglia and colleagues [26] reported that every minute of warm ischemia could diminish the postoperative kidney function; moreover, they identified 25 min as a safe cut-off for WIT in laparoscopic PN. In line with previous results, WIT > 25 min was a predictive factor for AKI in patients with and without DM.

Previous studies showed that male sex is a risk factor for AKI after PN as well as general surgery [10,12,14,20,27]. Consistent with prior results, we observed a significantly higher risk of postoperative AKI among male patients with and without DM. Sexual dimorphism exists in renal I/R injury, and sex hormones (i.e., ratio of testosterone to estrogen) are considered as the primary factor. Park and colleagues [28] showed that the presence of testosterone rather than the absence of estrogen is crucial in sex difference with respect to susceptibility to renal I/R injury via an increase in inflammation and functional injury to the kidney. Moreover, estrogen displayed a protective effect against renal I/R injury via activation of nitric oxide synthases, inhibition of endothelin-1 production, and depression of the renal sympathetic nervous system [28,29,30]. Thus, the combination of male sex and DM might have resulted in synergistic effects, and this might have led to our result that male patients with DM had approximately 14 times higher risk of developing AKI than female patients without DM.

HbA1c measurement is a standard method for assessing blood glucose management in patients with DM and it reflects the average blood glucose level over the past 2–3 months. The patients with an HbA1c level of 7% or more had a significantly increased risk of renal failure as well as cerebrovascular accidents, wound infection, and hospital death after coronary artery bypass surgery [31]. However, no study has determined whether elevated preoperative HbA1c increases AKI after PN. Our study is the first to demonstrate that preoperative HbA1c >7% was associated with increased risk for AKI after minimally invasive PN in patients with DM.

The present study has limitations, its main drawback being its retrospective observational nature; thus, it is susceptible to bias and other confounding factors. The baseline difference between patients with and without DM can confound analyses of AKI incidence and risk factors for AKI. In fact, prior to matching, the number of patients with DM who were older and had hypertension, cerebrovascular disease, and coronary artery disease was higher than that of patients without DM; however, these data were adjusted after propensity score matching. Although this study cannot replace a randomized trial, propensity score matching is a powerful tool for adjusting for confounding variables and reducing selection bias [32]. Therefore, this study is valuable in that it is the first study to compare patients with and without DM with respect to postoperative AKI using propensity score matching. Second, long-term functional outcomes of patients with AKI were not evaluated in the current study. Since postoperative AKI is known as a high-risk factor for new-onset chronic kidney disease [19], long-term follow-up studies are required.

In conclusion, patients with DM had an increased risk of developing AKI after minimally invasive PN, even after adjustment for other factors. Male patients with DM were most susceptible to AKI. WIT >25 min and preoperative HbA1c >7% were associated with AKI in patients with DM. Therefore, caution should be taken to reduce WIT during minimally invasive PN, especially in male patients with DM combined with elevated preoperative HbA1c.

## Figures and Tables

**Table 1:** Univariate and multivariate analyses of risk factors for acute kidney injury after minimally invasive partial nephrectomy (*N* = 884).

Variables	Univariate		Multivariate	
	OR (95% CI)	*P* value	OR (95% CI)	*P* value
Age, year	0.99 (0.98–1.01)	0.292		
Male sex	3.84 (2.51–5.87)	<0.001	4.57 (2.40–8.72)	<0.001
Body mass index, kg/m^2^	1.02 (0.99–1.05)	0.151		
ASA physical status				
I	1			
II	0.95 (0.66–1.36)	0.772		
III	1.39 (0.74–2.63)	0.309		
Co-morbidities				
Diabetes mellitus	2.56 (1.68–3.92)	<0.001	2.85 (1.71–4.74)	<0.001
Hypertension	1.39 (0.99–1.95)	0.056	1.10 (0.73–1.65)	0.654
Cerebrovascular disease	1.19 (0.43–3.27)	0.738		
Coronary artery disease	2.20 (0.92–5.28)	0.077	1.39 (0.52–3.69)	0.507
Preoperative lab value				
Creatinine, mg/dL	7.07 (2.78–18.03)	<0.001	0.79 (0.20–3.09)	0.734
Hematocrit, %	1.06 (1.02–1.10)	0.002	1.00 (0.95–1.05)	0.912
Hemoglobin A1c				
≤7%	1			
>7%	1.63 (0.76–3.50)	0.214		
Type of operation				
Laparoscopic	1			
Robotic	0.98 (0.67–1.44)	0.907		
Operation time, 60 min increase	1.44 (1.30–1.61)	<0.001	1.26 (1.08–1.47)	0.003
Warm ischemia time				
≤25 min	1		1	
>25 min	3.25 (2.30–4.58)	<0.001	2.81 (1.92–4.10)	<0.001
Intraoperative I & O				
Fluid input, 500 mL increase	1.46 (1.30–1.65)	<0.001	1.09 (0.91–1.31)	0.349
Colloid administration	1.22 (0.88–1.70)	0.238		
RBC transfusion	2.61 (1.28–5.32)	0.008	1.72 (0.71–4.19)	0.230
Urine output, 100 mL increase	1.06 (1.02–1.10)	0.002	1.06 (1.02–1.11)	0.008
Blood loss, 300 mL increase	1.55 (1.33–1.80)	<0.001		

OR—odds ratio; CI—confidence interval; ASA—American Society of Anesthesiologists; RBC—red blood cells; I & O—input and output.

**Table 2:** Demographic characteristics after propensity score matching.

Variables	After Case Matching (N = 404)			Before Case Matching (N = 884)		
	DM (N = 101)	Non–DM (N = 303)	*P* value	DM (N = 116)	Non–DM (N = 768)	*P* value
Age, year	58.7 (9.2)	58.8 (9.6)	0.887	60.0 (9.8)	51.5 (12.5)	<0.001
Male sex	69 (68%)	182 (60%)	0.139	78 (67%)	472 (61%)	0.231
Body mass index, kg/m^2^	26.8 (11.7)	25.0 (3.2)	0.131	26.6 (11.0)	24.6 (3.6)	0.065
ASA physical status			<0.001			<0.001
I	0	49 (16%)		0	295 (38%)	
II	91 (90%)	223 (74%)		100 (86%)	427 (56%)	
III	10 (10%)	31 (10%)		16 (14%)	46 (6%)	
Co-morbidities						
DM with oral medication	98 (97%)			113 (97%)		
DM with insulin	3 (3%)			3 (3%)		
Hypertension	68 (67%)	206 (68%)	0.902	83 (72%)	234 (30%)	<0.001
Cerebrovascular disease	4 (4%)	13 (4%)	>0.999	7 (6%)	15 (2%)	0.018
Coronary artery disease	4 (4%)	6 (2%)	0.276	8 (7%)	15 (2%)	0.006
Preoperative lab value						
Creatinine, mg/dL	0.8 (0.2)	0.8 (0.2)	0.821	0.8 (0.2)	0.8 (0.2)	0.530
Hematocrit, %	42.2 (5.1)	42.2 (4.5)	0.988	41.9 (5.1)	42.4 (4.4)	0.262
Hemoglobin A1c, %	7.3 (1.3)			7.3 (1.4)		
Type of operation			0.604			0.478
Laparoscopic	29 (29%)	79 (26%)		31 (27%)	182 (24%)	
Robotic	72 (71%)	224 (74%)		85 (73%)	586 (76%)	
Operation time, min	289.5 (78.3)	287.5 (98.0)	0.838	288.2 (78.2)	284.9 (91.2)	0.676
Warm ischemia time			0.952			0.511
≤25 min	64 (63%)	191 (63%)		72 (62%)	452 (59%)	
>25 min	37 (37%)	112 (37%)		44 (38%)	316 (41%)	
Intraoperative I & O						
Fluid input, mL	1793.5 (652.6)	1845.2 (658.8)	0.494	1812.9 (640.1)	1853.6 (652.8)	0.531
Patients administered with colloid, n	44 (44%)	125 (41%)	0.684	51 (44%)	326 (42%)	0.758
Patients transfused with RBC, n	7 (7%)	9 (3%)	0.135	10 (9%)	24 (3%)	0.009
Urine output, mL	583.7 (361.4)	563.7 (393.7)	0.652	609.6 (442.3)	593.9 (422.1)	0.710
Blood loss, mL	286.4 (337.7)	254.9 (303.4)	0.380	294.3 (336.8)	245.0 (288.6)	0.137

Values are presented as mean (SD) or number of patients (%). DM—diabetes mellitus; ASA—American Society of Anesthesiologists; RBC—red blood cells; I & O—input and output.

**Table 3:** Univariate and multivariate analyses of risk factors for acute kidney injury after minimally invasive partial nephrectomy in patients with and without DM.

Variables	DM (N = 101)				Non–DM (N = 303)			
	Univariate		Multivariate		Univariate		Multivariate	
	OR (95% CI)	*P*-value	OR (95% CI)	*P*-value	OR (95% CI)	*P*-value	OR (95% CI)	*P*-value
Age, year	1.00 (0.96–1.05)	0.947			0.99 (0.96–1.03)	0.581		
Male sex	10.87 (2.41–49.18)	0.002	19.58 (2.47–155.35)	0.005	4.89 (2.00–11.99)	0.001	4.52 (1.32–15.48)	0.016
Body mass index, kg/m^2^	0.99 (0.96–1.04)	0.973			1.06 (0.96–1.17)	0.236		
ASA physical status								
I					1			
II	1				0.51 (0.24–1.12)	0.093		
III	2.50 (0.67–9.36)	0.174			0.38 (0.10–1.51)	0.170		
Co-morbidities								
Hypertension	2.03 (0.77–5.35)	0.154			1.10 (0.55–2.22)	0.787		
Cerebrovascular disease	0.74 (0.07–7.45)	0.802			1.10 (0.24–5.16)	0.900		
Coronary artery disease	7.39 (0.74–74.13)	0.089	10.41 (0.79–136.42)	0.074	0.45 (0.02 –10.23)	0.616	0.39 (0.02–9.25)	0.557
Preoperative lab value								
Creatinine, mg/dL	21.28 (1.36–332.47)	0.029	5.46 (0.14–219.15)	0.367	7.37 (1.26–43.09)	0.027	0.25 (0.02–3.50)	0.301
Hematocrit, %	1.08 (0.99–1.18)	0.090	0.94 (0.83–1.08)	0.391	1.09 (1.02–1.18)	0.017	1.04 (0.95–1.13)	0.418
Hemoglobin A1c								
≤7%	1							
>7%	2.11 (0.89–5.01)	0.090	4.59 (1.47–14.36)	0.009				
Type of operation								
Laparoscopic	1				1			
Robotic	1.57 (0.59–4.19)	0.367			1.03 (0.49–2.16)	0.937		
Operation time, 60 min increase	1.35 (0.97–1.88)	0.077	1.24 (0.75–2.05)	0.408	1.45 (1.21–1.74)	<0.001	1.18 (0.90–1.56)	0.235
Warm ischemia time								
≤25 min	1		1		1		1	
>25 min	2.49 (1.04–5.94)	0.040	3.57 (1.17–10.94)	0.026	3.09 (1.59–6.01)	0.001	2.56 (1.24–5.26)	0.011
Intraoperative I & O								
Fluid input, 500 mL increase	1.31 (0.95–1.81)	0.101	0.93 (0.60–1.44)	0.752	1.63 (1.29–2.05)	<0.001	1.21 (0.87–1.68)	0.251
Colloid administration	1.33 (0.57–3.10)	0.516			0.92 (0.48–1.78)	0.805		
RBC transfusion	1.77 (0.37–8.42)	0.474	4.28 (0.40–46.09)	0.230	5.23 (1.35–20.33)	0.017	5.13 (0.96–27.42)	0.056
Urine output, 100 mL increase	0.91 (0.80–1.05)	0.185			1.07 (0.99–1.15)	0.097		
Blood loss, 300 mL increase	1.58 (1.07–2.33)	0.023			1.56 (1.20–2.04)	0.001		

OR, odds ratio; CI, confidence interval; ASA, American Society of Anesthesiologists; RBC, red blood cells; I & O, input and output.

**Table 4:** Incidence and odds ratio of acute kidney injury after minimally invasive partial nephrectomy based on the presence of DM and sex.

	Total patients, n	AKI incidence, n (%)	OR (95% CI)	*P* value
Non-DM female	121	8 (6.6%)	1	
DM female	32	2 (6.3%)	0.95 (0.16–5.59)	0.958
Non-DM male	182	37 (20.3%)	4.12 (1.32–12.86)	0.015
DM male	69	29 (42.0%)	14.46 (4.62–45.25)	<0.001

Values are presented as number of patients (%). AKI, acute kidney injury; OR, odds ratio; CI, confidence interval; DM, diabetes mellitus.
